# VEGF‐E Attenuates Injury After Ischemic Stroke by Promoting Reparative Revascularization

**DOI:** 10.1111/ejn.70114

**Published:** 2025-04-25

**Authors:** Romain Menet, Leila Nasrallah, Maxime Bernard, Anne‐Sophie Allain, Ayman ElAli

**Affiliations:** ^1^ Department of Psychiatry and Neuroscience, Faculty of Medicine Université Laval Quebec City Quebec Canada; ^2^ Neuroscience Axis Research Center of CHU de Québec ‐ Université Laval Quebec City Quebec Canada

**Keywords:** cerebral ischemia, endothelial cells, pericytes, repair, therapeutic angiogenesis, VEGF‐E

## Abstract

The angiogenic response after stroke correlates with mild injury and an improved recovery. Stimulation of post‐stroke angiogenesis using vascular endothelial growth factor (VEGF)‐A is associated with an increased risk of vascular destabilization, leading to life‐threatening complications. The non‐mammalian VEGF‐A homolog, VEGF‐E, stimulates stable cutaneous vascularization and promotes wound healing. Herein, we posit that VEGF‐E represents a potential mediator of reparative revascularization after ischemic stroke. C57BL6/J wildtype mice were subjected to experimental stroke, and VEGF‐E or VEGF‐A were intranasally delivered during the subacute phase. Our results indicate that VEGF‐E improves neurological recovery and increases vascular density without compromising permeability, more efficiently than VEGF‐A. We show that VEGF‐E‐mediated revascularization correlates with normal restoration of brain perfusion, whereas VEGF‐A induces cerebral hyperperfusion, indicative of vascular dysfunction. Furthermore, VEGF‐E reduces microvascular stalls, increases the density of angiogenic vasculature, and improves the interaction of brain endothelial cell with pericytes, which is critical for vascular stabilization. Using cell‐based assays, we demonstrate that stimulation of brain endothelial cells with VEGF‐E, but not with VEGF‐A, increases the expression of platelet‐derived growth factor (PDGF)‐D, a potent ligand of PDGFRβ that plays critical roles in regulating the survival and functions of perivascular cells, including pericytes. These effects are associated with activation of extracellular signal‐regulated kinase (ERK)1/2 and P38 mitogen‐activated protein kinase (MAPK). Finally, we confirm that the secretome of VEGF‐E‐stimulated brain endothelial cells ameliorates pericyte migration required for vascular recruitment. Our study indicates that VEGF‐E promotes a stable and functional revascularization after ischemic stroke, outlining its promises for therapeutic purposes.

## Graphical abstract

VEGF‐E mediates a reparative revascularization after ischemic stroke. VEGF‐E subacute delivery into the brain via the intranasal route improves recovery via promotion of the formation of stable and functional vasculature by enhancing brain endothelial cell interaction with perivascular cells, including pericytes. VEGF‐A mediated‐revascularization after stroke is associated with a compromised stability and functionality.

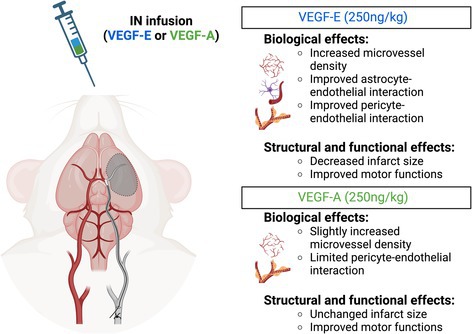

**Abbreviations**
ABC: avidin‐biotin complex
ANOVA: analysis of variance
AQP4: aquaporin‐4
BBB: blood–brain barrier
BCA: bicinchoninic acid
CCA: common carotid artery
CD: cluster of differentiation
CNS: central nervous system
DAB: diaminobenzidine
DAPI: 4′,6‐diamidino‐2‐phenylindole
DMEM: Dulbecco's modified Eagle's medium
DPX: distyrene plasticizer xylene
ECA: external carotid artery
ECL: enhanced chemiluminescence plus
ECM: endothelial cell medium
ECGS: endothelial cell growth serum
ERK: extracellular signal‐regulated kinase
EtOH: ethanol
FBS: fetal bovine serum
FJB: fluoro‐Jade B
GFAP: glial fibrillary acidic protein
HBVP: human brain vascular pericytes
HR: high resolution
HRP: horseradish peroxidase
IBA1: ionized calcium binding adaptor molecule
ICA: internal carotid artery
IgG: immunoglobulin G
iHBEC: immortalized human brain endothelial cells
KPBS: potassium phosphate‐buffered saline
LDF: laser Doppler flowmeter
LSCI: laser Speckle contrast imaging
MAPK: mitogen‐activated protein kinase
MCAo: middle cerebral artery occlusion
MMPs: matrix metalloproteases
NGS: normal goat serum
NIR: near infrared
NSS: neurological severity score
OGD: oxygen and glucose‐deprived
PB: phosphate buffer
PBS: phosphate‐buffered saline
PDGF: platelet‐derived growth factor
PDGFR: platelet‐derived growth factor receptor
PFA: paraformaldehyde
PGS: pericytes growth serum
PVDF: polyvinylidene fluoride
ROI: region of interest
RT: room temperature
SD: standard deviation
SDS: sodium dodecyl sulfate
SDS‐PAGE: sodium dodecyl‐sulfate polyacrylamide gel electrophoresis
TBS: tris‐buffered saline
TGF‐β1/3: transforming growth factor‐β1/3
tPA: tissue plasminogen activator
VEGF: vascular endothelial growth factor
VEGFR: vascular endothelial growth factor receptor

## 1 Introduction

Ischemic stroke represents a major cause of death and disability of the adults worldwide. It results from the interruption of blood supply into a specific brain region, leading to injury and neurological deficits (Dirnagl 2012). The current therapeutic options are limited to re‐canalization of the obstructed cerebral artery to restore the regional blood supply through tissue plasminogen activator (tPA)‐mediated thrombolysis or mechanical thrombectomy (Feske 2021). Unfortunately, still no disease‐modifying therapy exists. Initial injury followed by reperfusion jointly generates a heterogenous brain lesion comprising the infarct core that contains irreversibly lost tissue, surrounded by a peri‐infarct region comprising salvageable tissue (Sodaei and Shahmaei 2020). Compelling evidence is suggesting that the peri‐infarct region is actively attempting to repair itself via various reparative processes to promote neurological recovery (Cramer 2008; Yang et al. 2017). Maturation of definitive lesion narrowly depends upon the severity of initial acute injury and the progression of subacute secondary injury associated with neurovascular deregulations (Merali et al. 2017; Pillai et al. 2009). Attenuation of secondary injury progression would limit definitive lesion expansion.

Angiogenesis represents a key endogenous reparative response triggered at the peri‐infarct region to enable compensatory revascularization to meet the metabolic needs of the injured brain and to facilitate elimination of cell debris by immune cells (Eichmann and Thomas 2013; Kanazawa et al. 2019). The angiogenic response is regulated through the concerted action of pro‐angiogenic and angiostatic factors, implicating endothelial cell proliferation and migration to form new vascular network deriving from a pre‐existing one (Fan and Yang 2007; Marti et al. 2000). The coverage of nascent vasculature by perivascular cells, namely pericytes, and astrocyte endfeet constitutes a critical step for vascular stabilization and maintenance of blood–brain barrier (BBB) properties (Bernard et al. 2024; Cao et al. 2021; Pisani et al. 2022). The expression of vascular endothelial growth factor (VEGF)‐A, a potent pro‐angiogenic factor, increases after ischemic stroke (Babkina et al. 2022; Marti et al. 2000). Early reports indicated that endothelial cell proliferation at the injury site occurs as early as Day 1 post‐stroke, and vascular density increases 3 to 4 days post‐stroke in humans and rodents (Hayashi et al. 2003). VEGF‐A elevated levels correlate with higher vascular density that is associated with mild structural and neurological damage upon ischemic stroke (Li, Pan, Qin, et al. 2014). VEGF‐A mediates its biological effects by binding and activating VEGF receptor (VEGFR)‐2 expressed mainly in endothelial cells (Olsson et al. 2006; Shahriar et al. 2024). VEGF‐A binds as well to VEGFR‐1 that acts as decoy receptor in endothelial cells but as a signaling receptor in perivascular cells, glial cells, and immune cells (Jean LeBlanc, Guruswamy, and ElAli 2018; Uemura et al. 2021). These observations outline the promises of developing new therapeutic interventions that are based on promoting revascularization of the injured tissue to stimulate recovery (Y. Wang et al. 2007). Numerous investigations have assessed the exogenous administration of different recombinant forms of VEGF‐A, leading to mitigated results (Hermann and Zechariah 2009; Zechariah et al. 2013). Exogenous administration of VEGF‐A increases the risk of BBB breakdown and hemorrhagic transformation after stroke (Zhang et al. 2000). These detrimental effects are essentially driven by an excessive degradation of the extracellular matrix proteins and an altered interaction of brain endothelial cells with pericytes, leading to overall vascular destabilization (Valable et al. 2005).

Identification of novel mediators that enable stable and functional revascularization would allow achieving important breakthroughs in stroke therapies (Moon et al. 2021). Our group has previously demonstrated that rescuing the survival and functions of pericytes after ischemic stroke promotes recovery by enhancing vascular stability and functionality (Jean LeBlanc, Guruswamy, and ElAli 2018; Bernard et al. 2024). These findings provide new framework to develop efficient therapeutic interventions that emphasize on simultaneously stimulating the formation of new vasculature while enhancing the association of pericytes to brain endothelial cells. Previous reports have shown that VEGF‐E, a non‐mammalian VEGF‐A homolog encoded by the parapox Orf virus, promotes the formation of a stable cutaneous vasculature (Wise et al. 2003, 2018, 2020). VEGF‐E specifically binds to VEGFR‐2, but not VEGFR‐1, and potently promotes endothelial cell proliferation (Ogawa et al. 1998; Wise et al. 2003, 2018, 2020). Exogenous VEGF‐E promotes cutaneous wound healing by inducing vascularization in the absence of edema or recruitment of immune cells expressing VEGFR‐1 (Wise et al. 2018). Importantly, VEGF‐E enhances the vascular coverage by pericytes and attenuates the formation of excessive scar associated with improved collagen structure during the process of wound healing (Wise et al. 2018).

Herein, our study aims to investigate the therapeutic potential of VEGF‐E in promoting a stable and functional revascularization after stroke. For this purpose, recombinant VEGF‐E or VEGF‐A were delivered non‐invasively via the intranasal route into the brain of mice subjected to experimental ischemic stroke model via transient middle cerebral artery occlusion (MCAo). Our findings reveal that VEGF‐E attenuates secondary injury progression and neurological deficits by promoting stable and functional revascularization. In contrast to VEGF‐A, VEGF‐E improves vascular density without compromising permeability, associated with an enhanced coverage of pericytes. This outlines a synergistic regulation of vascular formation and stabilization. Mechanistically, VEGF‐E induces the expression of platelet‐derived growth factor (PDGF)‐D in brain by the endothelial cells, associated with activation of extracellular signal‐regulated kinase (ERK)1/2 and P38 mitogen‐activated protein kinase (MAPK). Endothelial PDGF‐D is a potent ligand of platelet‐derived growth factor receptor (PDGFR)β that promotes the survival and functions of pericytes after stroke (Bernard et al. 2024). Our results indicate that VEGF‐E potently mediates stable and functional revascularization after stroke.

## 2 Material and Methods

### 2.1 Animal Experiments

Three to 5 months old C57BL6/J male mice obtained from The Jackson Laboratory (Bar Harbor, ME, USA) were used. Mice were housed and acclimated to standard laboratory conditions (12 h light/dark cycle; lights on at 7:00 AM and off at 7:00 PM) with free access to chow and water ad libitum (Bernard et al. 2024). Mice were subjected to focal ischemic stroke via transient MCAo, as described (Bernard et al. 2024). Briefly, mice were anesthetized with 3% isoflurane for induction and 1.5% isoflurane for maintenance (95% O_2_, 2 L/min) and body temperature was maintained between 36°C and 37°C using a feedback‐controlled heating system (Harvard Apparatus®, QC, Canada) throughout the procedure. After midline neck incision, left common carotid artery (CCA) and external carotid artery (ECA) were isolated and ligated. A microvascular clip was placed on the internal carotid artery (ICA), and a 7–0 silicon‐coated nylon monofilament (Doccol Corporation, MA, USA) was directed through the ICA until the origin of MCA. The monofilament was left in place for 45 min and reperfusion was achieved by gently withdrawing it. Laser Doppler flowmeter (LDF) (OMEGAFLOW FLO‐C1; Omegawave Inc., Japan) was used to monitor brain perfusion using a flexible fiber optic probe attached to the skull overlying the MCA territory to confirm occlusion (Bernard et al. 2024). All animal procedures and handling were performed according to the Canadian Council on Animal Care guidelines, as implemented by *Comité de Protection des Animaux de l'Université Laval‐3* (CPAUL‐3; Protocol # 19‐063). Animal studies were reported according to ARRIVE 2.0 guidelines.

### 2.2 VEGF‐E and VEGF‐A Intranasal Delivery

Recombinant VEGF‐E (NZ‐7; PromoCell, ON, Canada; C‐64416 or Mybiosource, CA, USA; MBS142140) or recombinant VEGF‐A_165_ (Thermo Fischer Scientific; QC, Canada; 100‐20) were resuspended in sterile water and delivered non‐invasively into the brain through the intranasal route, as previously described (Jean LeBlanc, Guruswamy, and ElAli 2018; Bernard et al. 2024). Briefly, 30 min prior to intranasal infusion, mice (*n* = 5–7 animals per group) were intranasally treated with 15 μL hyaluronidase (100 U; Sigma‐Aldrich, ON, Canada) to improve its absorption and subsequent delivery into the brain via permeabilization of the olfactory epithelium (Jung 2020). Mice received 15 μL intranasal infusion of either vehicle (VEH; saline), VEGF‐A (250 ng/kg), or VEGF‐E (250 ng/kg), starting at 24 h after MCAo, repeated at 72 h, and were euthanized 4 days after stroke.

### 2.3 Brain Perfusion Imaging and Analysis

Laser Speckle contrast imaging (LSCI) was used to visualize and measure the temporal changes in brain perfusion, as previously described (Bernard et al. 2024). Prior to imaging, mouse head was shaved and a 100 μL of lidocaine/bupivacaine solution was applied on the incision site and a 50 μL on the ears, followed by disinfection of skin. Mice were placed on a stereotaxic frame (RWD Life Science Inc., CA, USA) and were anesthetized using 1.5% isoflurane (95% O_2_, 2 L/min). The skull was exposed using fine‐tip forceps and stabilized at 10 cm distance of the camera of 2D laser Speckle blood flow imager (OMEGAZONE OZ‐3, Omegawave Inc.). The OZ‐3 system is equipped with a visible and near infra‐red (NIR) CCD camera, which allows continuous visualization of the real images and comparison with blood flow images. Continuous colored blood flow images were recorded at high resolution (HR) mode using a built‐in measurement software and analyzed using a built‐in software that enables simultaneous quantification of relative blood flow in different region of interests (ROI)s consistently placed at the brain region irrigated by the MCA in the ipsilateral and contralateral hemisphere. The relative brain perfusion was measured in the same animals at 5 min and 4 days after stroke in each hemisphere and was calculated by averaging 20 consecutive raw Speckle images in each of the ROIs. A brain perfusion ratio of the ipsilateral over contralateral hemisphere was calculated and presented as a ratio of 4 days over 5 min to reflect the temporal changes.

### 2.4 Neurobehavioral Analysis

*The pole test* was used to assess overall locomotor function after stroke. Briefly, a mouse was placed on the top of a 60‐cm vertical pole with a rough surface, which has a diameter of 10 mm. Time taken to reach the ground was recorded. If the mouse stopped during a run, then the trial was excluded and repeated (Li, Zhang, Lin, et al. 2014). If the animal was unsuccessful in performing the task by falling or sliding, a score of 20 s was accorded, which was considered as the maximum time. Each animal was tested prior to MCAo and were next randomly assigned to an experimental group. Animals were tested 24 h and 4 days after MCAo. For each time point, each animal was tested three times, and the average was used for statistical analysis.

*Neurological severity scores* were computed at 24 h and 4 days after MCAo induction, as previously described (Jean LeBlanc et al. 2019). The sensorimotor performance of mice subjected to MCAo was assessed using a neurological score test that closely correlates neurological deficits with the severity of histological brain damage. The neurological deficits were evaluated using the following score: 0 = normal function; 1 = mild circling behavior with attempts to rotate to the contralateral side upon lifting of the animal by the tail; 2 = circling to the contralateral side but normal posture at rest; 3 = reclination and consistent circling to the contralateral side at rest, with mouse nose almost reaching its tail; and 4 = absence of spontaneous motor activity.

### 2.5 Brain Tissue Processing

For histochemical and immunohistochemical analyses, mice were euthanized via a transcardiac perfusion with 0.1 M phosphate‐buffered saline (PBS) followed by 1% paraformaldehyde (PFA) fixation. Brains were retrieved and post‐fixed in 4% PFA for 24 h and transferred into 4% PFA containing 20% sucrose for 24 h at 4°C. Brains were then cut into 25‐μm coronal sections using a freezing microtome (Leica Biosystems, ON, Canada), serial sections were collected in a 12‐well plates filled with an anti‐freeze solution (30% glycerol, 30% ethylene glycol in 0.9% NaCl, phosphate buffer [PB]) and kept at −20°C until further use.

### 2.6 Analysis of Injury Size and BBB Permeability

Immunohistochemical analysis was used to assess the extravasation of blood‐borne immunoglobulin G (IgG) into the brain, an endogenous marker of major BBB breakdown, as previously described (ElAli et al. 2011). Briefly, PFA‐fixed brain sections were washed with 0.1 M potassium PBS (KPBS) (Sigma‐Aldrich, MO, USA) and then incubated for 20 min in a permeabilization/blocking solution containing 4% normal goat serum (NGS), 1% BSA (Sigma‐Aldrich), and 1% Triton X‐100 (Sigma‐Aldrich) in 0.1 M KPBS. Next, sections were incubated with a biotinylated anti‐mouse IgG antibody (1/1000; Vector laboratories; BA‐9200) overnight at 4°C. The signal was revealed using avidin peroxidase kit (Vectastain Elite; Vector Labs, CA, USA) by immersing the sections for 1 h in avidin‐biotin complex (ABC) mixture. Brain sections were washed, stained in diaminobenzidine (DAB) solution for 10 min and rinsed 3× for 5 min in 0.1 M KPBS. DAB‐stained brain sections were mounted onto SuperFrost® Plus slides (Fisher Scientific, ON, Canada), dried overnight under vacuum at room temperature (RT), dehydrated, and finally cover‐slipped with distyrene plasticizer xylene (DPX; Electron Microscopy Sciences, PA, USA). Finally, brain sections were digitized and analyzed for areas exhibiting IgG extravasation using FIJI software (National Institutes of Health, MD, USA). IgG extravasation was represented as percent of fold change of the contralateral *((contralateral − intact ipsilateral) / contralateral) × 100)*. Edema size was represented as percent of fold change of the contralateral *((ipsilateral / contralateral) × 100)*.

### 2.7 Fluoro‐Jade B (FJB) Staining

FJB staining was used to assess neuronal degeneration, as described (Lecordier et al. 2023). Briefly, free‐floating PFA‐fixed brain sections were washed with 0.1 M KPBS 3× for 10 min, then mounted onto Superfrost® Plus slides and dried overnight at RT. Mounted brain sections were fixed with 4% PFA for 20 min, then rinsed twice with 0.1 M KPBS for 5 min and processed through a cycle of dehydration/rehydration in ethanol (EtOH) (3 min in 50%, 1 min in 70%, 3 min in 100%, 1 min in 70%, 1 min in 50%, and 1 min in distilled water). Mounted brain sections were next treated for 10 min with 0.06% potassium permanganate (MP Biomedicals, CA, USA), rinsed for 1 min with distilled water, and then incubated in 0.2% FJB solution (EMD Millipore, ON, Canada) containing 0.1% acetic acid + 0.0002% 4′,6‐diamidino‐2‐phenylindole (DAPI) in milliQ water for 10 min. Sections were rinsed in milliQ water and dried overnight, then immersed 3× for 2 min in xylene and cover‐slipped with DPX. Density of FJB^+^ cells was quantified using unbiased computer‐assisted stereological software (Stereologer; SRC Biosciences, FL, USA).

### 2.8 Immunofluorescence Analysis

Free‐floating PFA‐fixed brain sections were rinsed 3× with 0.1 M KPBS and incubated at RT in a permeabilization/blocking solution containing 4% NGS, 1% BSA, and 1% Triton X‐100 in 0.1 M KPBS for 45 min. Brain sections were then incubated with primary antibodies diluted in the permeabilization/blocking solution overnight at 4°C. The following primary antibodies were used: rat anti‐mouse cluster of differentiation (CD31) (1/500; BD Biosciences, ON, Canada; 550274), goat anti‐mouse aminopeptidase N (CD13) (1/250; R&D systems), rat anti‐mouse glial fibrillary acidic protein (GFAP) (1/500; Invitrogen, MA, USA; 13‐0300), rabbit anti‐mouse ionized calcium binding adaptor molecule (IBA1) (1/500; WAKO, ON, Canada; 019‐19741), goat anti‐rat CD45 (1/500; BD Biosciences; 553076), rabbit anti‐mouse PDGFRβ (1/250; Abcam, ON, Canada; ab32570), rat anti‐mouse endoglin (CD105) (1/250; Novus Biologicals, ON, Canada; NB100‐77666), and rabbit anti‐mouse aquaporine‐4 (AQP4) (1/500; Cell Signaling Technology, MA, USA; 59678S). The next day, brain sections were rinsed 3× with 0.1 M KPBS and incubated for 2 h at RT in the dark with one the following secondary antibodies at a dilution of 1/1000: Alexafluor 546 goat anti‐rat (Invitrogen; A11081), Alexafluor 546 donkey anti‐goat (Invitrogen; A11056), and Cy5 goat anti‐rabbit (Invitrogen; A10523). Brain sections were then rinsed 2× with 0.1 M KPBS, incubated with DAPI (1/10000; Invitrogen) for 5 min, mounted onto Superfrost® Plus slides, and cover‐slipped with Fluoromount‐G® anti‐fade medium (Electron Microscopy Sciences). Epifluorescence images were acquired using Axio Observer microscope equipped with a module for optical sectioning (Apotome.2) and Axiocam 503 monochrome camera and then processed using ZEN Imaging Software (Carl Zeiss Canada, ON, Canada). The density of microvessels, pericytes, astrocyte endfeet, and microvascular stalls was measured using FIJI software in four regions of interests (ROIs; 450 μm × 350 μm) per animal randomly distributed in the injured striatum of cortex, acquired with a 20× objective, as described (Jean LeBlanc et al. 2019). Vascular coverage of pericytes (CD13 or PDGFRβ) and astrocyte endfeet (AQP4) was represented as percent of density in endothelial cells (CD31 or CD105).

### 2.9 Human Brain Endothelial and Perivascular Cell Culture

Immortalized human brain endothelial cells (iHBEC) (Cedarlane Laboratories, ON, Canada; CRL‐3245; 70041474) were used to investigate the molecular mechanisms underlying VEGF‐E action. iHBEC cells have all the characteristics needed to represent the BBB and interact with pericytes (Chen et al. 2018; Zolotoff et al. 2022). For iHBEC, the surface of flasks/wells was pre‐coated with 0.1% gelatin diluted in water (STEMCELL Technologies, bc, Canada) for at least 1 h before iHBEC were seeded. iHBEC were cultured at 37°C in 5% CO_2_, 95% air in endothelial cell medium (ECM; ScienCell Research Laboratories, CA, USA) containing 2% FBS, 100 U/mL endothelial cell growth serum (ECGS), and 100 U/mL streptomycin/penicillin. Moreover, primary human brain vascular pericytes (HBVP) (ScienCell; 1200; 33634) were used to investigate the action of VEGF‐E on factor release from iHBEC cells. HBVP were cultured at 37°C in 5% CO_2_, 95% air in a Dulbecco's modified Eagle's medium (DMEM) containing 4.5 g/L glucose (Multicell; Wisent, QC, Canada) containing 2% fetal bovine serum (FBS), 100 U/mL pericytes growth serum (PGS), and 100 U/mL streptomycin/penicillin. In all experiments, cells were grown to approximately 80% confluence and subjected to a maximum of seven passages. Cells were treated with saline or 100 ng/mL of VEGF‐E (NZ‐7; PromoCell or Mybiosource) or VEGF‐A_165_ (Thermo Fisher Scientific). At the end of each experiment, cell culture medium was collected, and cells were harvested for protein extraction.

### 2.10 Oxygen and Glucose Deprivation

To investigate the responses of iHBEC to ischemia and reperfusion‐like conditions, cells were incubated under oxygen and glucose‐deprived (OGD) condition, as previously described (Jean LeBlanc et al. 2019; Lange et al. 2016). iHBEC were seeded at 3 × 10^5^ cells/well, in a 12‐well plate (Corning, NY, USA). OGD was induced by incubating cells at 37°C in DMEM‐glucose free medium (Multicell; Wisent) under hypoxic condition (1% O_2_, 5% CO_2_) for 40 h using a hypoxia chamber (STEMCELL Technologies) to generate ischemia‐like condition. After OGD induction, DMEM‐glucose free medium was replaced by ECM glucose‐normal medium, and cells were incubated under normal oxygenation condition, mimicking the effect of reperfusion, with or without VEGF‐E at 100 ng/mL for 24 h. As control, cells were incubated at 37°C in ECM glucose‐normal medium (ScienCell) under normal oxygen condition (normoxia). Cells were harvested for protein extraction.

### 2.11 Western Blot Analysis

iHBEC were lysated using NP40 lysis buffer supplemented with 1% protease inhibitor cocktail (Sigma‐Aldrich) and 1% phosphatase inhibitor cocktail (Sigma‐Aldrich) (Jean LeBlanc, Guruswamy, and ElAli 2018). Total protein concentration in each sample was determined using bicinchoninic acid (BCA) (QuantiPro Assay Kit; Sigma‐Aldrich) (Jean LeBlanc, Guruswamy, and ElAli 2018). Protein samples (20 μg) were mixed with 2× sodium dodecyl sulfate (SDS)‐loading buffer and heated for 10 min at 95°C. Samples were run on 10%, 12%, or 14% polyacrylamide gel electrophoresis (SDS‐PAGE) and subjected to electrophoresis using Mini‐PROTEAN® Tetra Cell (Bio‐Rad, Hercules, CA, USA). After migration, resolved protein bands were transferred onto a 0.45‐μm polyvinylidene fluoride (PVDF) membrane (EMD Millipore) for 10 min at 25 V using Trans‐Blot® Turbo™ Transfer System (Bio‐Rad). The PVDF membranes were rinsed 3× for 10 min with 0.1 M Tris‐buffered saline (TBS) solution containing 0.5% Tween‐20 (TBS‐T; Sigma‐Aldrich) and blocked with skim milk in TBS‐T 5% (w/v) for 30 min at RT. The PVDF membranes were then incubated overnight at 4°C with the following primary antibodies diluted at 1/1000 in TBS‐T solution: rabbit anti‐extracellular signal‐regulated kinase (ERK)1/2 (Cell Signaling; 9102), rabbit anti‐PDGF‐D (Abcam; ab181845), rabbit anti‐Thr202/Tyr204 ERK1/2 (p‐ERK1/2; Cell Signaling; 9101), rabbit anti‐P38 mitogen‐activated protein kinase (MAPK) (Cell Signaling; 92125), rabbit anti‐Thr180/Tyr182 P38 (p‐P38; Cell Signaling; 9211), mouse anti‐α‐tubulin (Santa Cruz; sc‐32293), and mouse anti‐actin (EMD Millipore; MAB1501). Primary antibodies were detected with horseradish peroxidase (HRP)‐conjugated secondary antibodies (Jackson ImmunoResearch Laboratories Inc. USA) diluted at 1/10000 in TBS‐T and revealed by enhanced chemiluminescence plus (ECL) solution (Bio‐Rad). Actin and α‐tubulin were used to ensure equal protein loading. Blots were digitized using Biorad chemidoc XRS^+^ system (Bio‐Rad) and were densitometrically analyzed using FIJI software corrected for protein loading by means of either actin or α‐tubulin, and expressed as relative values (Jean LeBlanc et al. 2019).

### 2.12 Wound Healing Assay

The wound healing assay was used to assess the migratory capacity of HBVP. Briefly, 3 × 10^5^ HBVP/well (three replicates per condition) were plated into a 12‐well plate and incubated under the appropriate condition to reach confluence. The cell monolayer was scratched at well midline using a 1 mm pipette tip and washed with serum‐free medium to remove detached cells. HBVP were then cultured in complete medium supplemented with vehicle (VEH; PBS), VEGF‐E (100 ng/mL), or with the conditioned media of iHBEC stimulated with VEGF‐E (medium composed of half‐conditioned media and half HBVP media), under normoxic conditions. Brightfield images were acquired using the 10× objective of Axio Observer microscope (Carl Zeiss, Canada) immediately after scratching cells (0 h), 24 h, and 48 h later. Cell migration was assessed by quantifying wound closure, calculated as follows: *((A*_*0*_ *− A*_*n*_ *) / A*_*0*_ *× 100)*, where *A*_*0*_ represents initial complete area of the wound and *A*_*n*_ represents the remaining open area of the wound.

### 2.13 Point Pattern Analysis (PPA)

We identified individual CD45^+^ and IBA1^+^ cells in the ischemic hemisphere using a custom‐made script (groovy) for QuPath. The cells' coordinates were batch‐processed in R‐statistical software using the *statspat* package to generate hyper‐frames containing point patterns (ppp). Using the *rhohat* function from *spatstat*, we calculated individual nonparametric estimates of the marker's spatial intensity with x‐coordinates as a covariant. The resulting *fv* objects were then pooled by condition using the *pool* function, obtaining mean estimates and 95% confidence intervals over the whole observation window. We fitted a logarithmic fixed effects point process models using the *mppm* function from *spatstat* to regress the spatial intensity on condition. Results are presented in logarithmic scale. All R code used to produce PPA analyses are available at https://github.com/elalilab/VEGF‐E‐stroke_ElAli

### 2.14 Statistical Analysis

Results are presented as boxplot with min/max or line chart ± standard deviation (SD). Data distribution normality was assessed using the Shapiro–Wilk test. For comparisons between two groups, unpaired tow‐tailed *t*‐test was used. For multiple comparison, one‐way or two‐way analysis of variance (ANOVA) followed by Tukey's post‐hoc test was used. For data not passing the normality test, Mann–Whitney test or Kruskal–Wallis followed by Dunn's multiple comparisons test were used. *p* < 0.05 was considered statistically significant. Statistical analyses were carried out using GraphPad Prism Version 10.0 for OS X (GraphPad Software, CA, USA).

## 3 Results

### 3.1 Subacute VEGF‐E Brain Delivery Promotes Recovery and Alleviates Structural Damage

**FIGURE 1:**
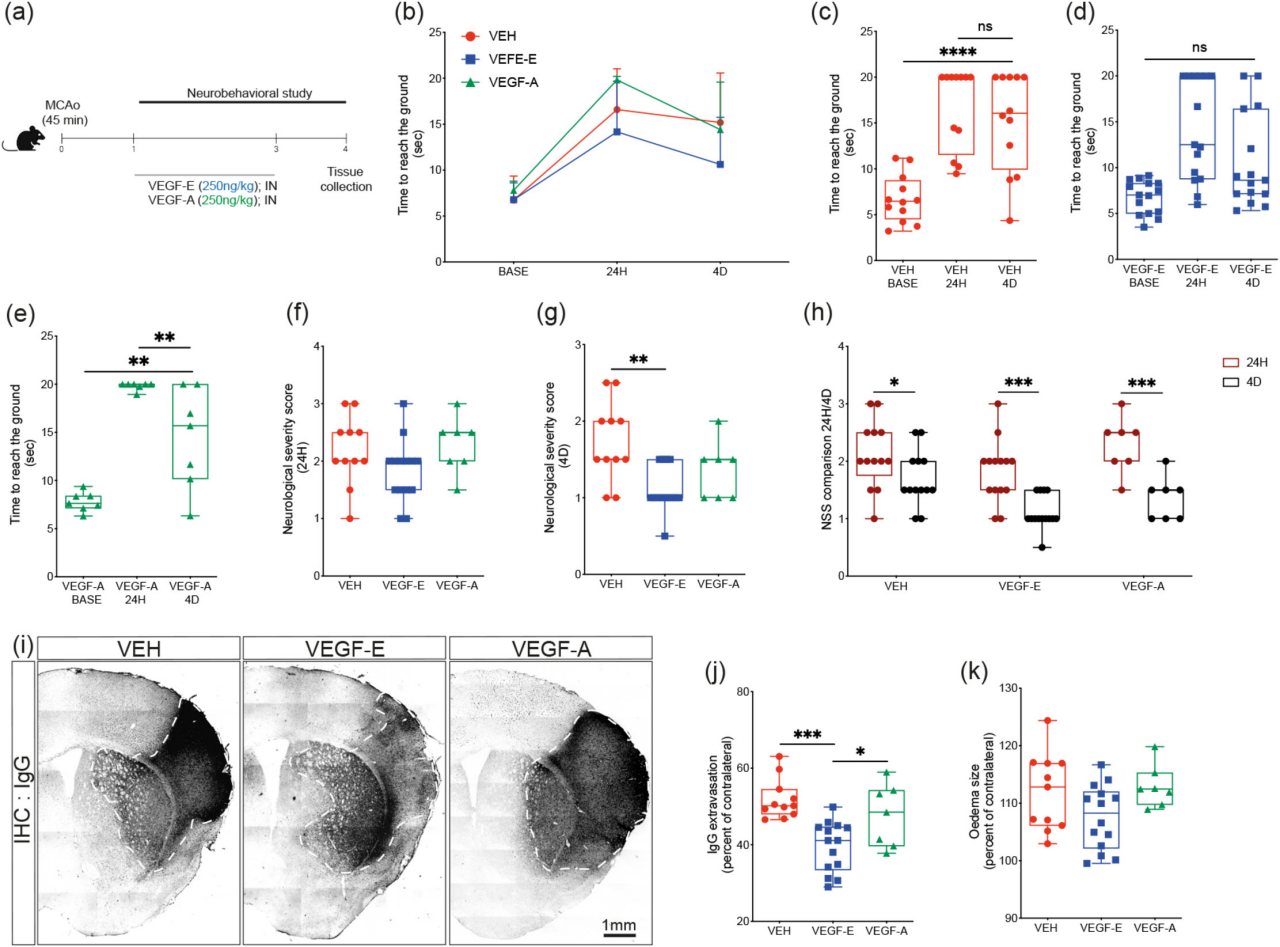
Motor deficits and structural damage are attenuated by VEGF‐E. (a) Schematic illustration of the experimental design of VEGF‐E and VEGF‐A intranasal delivery initiated at 24 h and repeated at Day 3 in mice subjected to ischemic stroke using MCAo, followed by euthanasia at Day 4. (b) Intergroup analysis of the pole test in vehicle (VEH)‐, VEGF‐E‐, and VEGF‐A‐treated mice at baseline, 24 h, and 4 days after stroke. Analysis of the pole test at baseline, 24 h, and 4 days after stroke in (c) VEH‐, (d) VEGF‐E‐, and (e) VEGF‐A‐treated mice. Analysis of the neurological severity score at (f) 24 h and (g) 4 days after stroke. (h) Intragroup analysis of the neurological severity score at 24 h and 4 days after stroke. (i) Representative images of IgG immunohistochemistry showing extravasation that reflects vascular permeability and injury severity (dark area surrounded by white dashed line) in the ipsilateral hemisphere. (j) Analysis of IgG extravasation represented as percent of contralateral hemisphere in VEH‐, VEGF‐E‐, and VEGF‐A‐treated mice. (k) Analysis of brain edema represented as percent of contralateral hemisphere in VEH‐, VEGF‐E, and VEGF‐A‐treated mice. Data are boxplot with min/max (*n* = 5–7 animals/group) or line plot superimposed symbols at mean with connecting lines. *p < 0.05, **p < 0.01, ***p < 0.001, ^****^ *p* < 0.0001 compared to control (c, e, g, h, j, one‐way ANOVA). Abbreviations: ANOVA, analysis of variance; BASE, baseline; D, days; H, hours; IHC, immunohistochemistry; IN, intranasal; VEGF, vascular endothelial growth factor; VEH, vehicle.

VEGF signaling is activated in the early phases of ischemic stroke at the injury site, correlating with an active angiogenic response that limits neuronal loss (Fang, Wang, and Miao 2023; Geiseler and Morland 2018). Exogenous VEGF‐A increases the risk of exacerbating vascular permeability (Corti et al. 2022; Geiseler and Morland 2018). Herein, we aimed to explore the potential of VEGF‐E in promoting stable revascularization after stroke and in attenuating secondary injury progression in the subacute phase. For this purpose, VEGF‐E or VEGF‐A were intranasally infused 24 h after MCAo induction in C57BL6/J mice, repeated at 72 h to ensure its bioavailability, and mice were euthanized at Day 4 (Figure 1a). Our strategy aims to target the subacute phase during which the angiogenic response is active. First, different neurobehavioral paradigms were employed to assess the impact of subacute delivery of VEGF‐E and VEGF‐A on motor functions. In the pole test, the time to reach the ground among the different groups was similar (Figure 1b). However, when looking into each group individually over time, we found that VEH‐ and VEGF‐A‐treated mice have a slower functional locomotor recovery compared to VEGF‐E‐treated mice (VEH BASE vs. VEH 4D, *p* < 0.0001; VEGF‐A BASE vs. VEGF‐A 4D, *p* = 0.0015; VEGF‐E BASE vs. VEGF‐E 4D; *p* = 0.2443) (Figure 1b–e). This outlines a more efficient recovery of locomotion in VEGF‐E‐treated mice. Furthermore, neurological severity score indicated that sensorimotor deficits remained unchanged 24 h after stroke among all groups (Figure 1f), whereas VEGF‐E‐treated mice exhibited attenuated neurological deficits at Day 4 (Figure 1g). Notably, neurological severity score was slightly attenuated between 24 h and 4 days in the VEH‐treated mice (*p* = 0.0268), whereas it was markedly reduced in VEGF‐E‐ (*p* = 0.0002) and VEGF‐A‐ (*p* = 0.0005) treated animals (Figure 1h). These observations suggest that sensorimotor deficits were more efficiently attenuated over time upon VEGF‐E intranasal infusion in comparison to VEGF‐A. Analysis of IgG immunohistology (Figure 1i) indicated that VEGF‐E attenuated IgG extravasation (Figure 1j), more efficiently compared to VEGF‐A, outlining a reduced permeability at Day 4 after stroke, reflected by the unchanged edema size (Figure 1k). These findings indicate that VEGF‐E subacute delivery improves motor functions without compromising vascular permeability.

### 3.2 VEGF‐E Enhances Vascular Density and Functionality to Enable Proper Brain Perfusion

**FIGURE 2:**
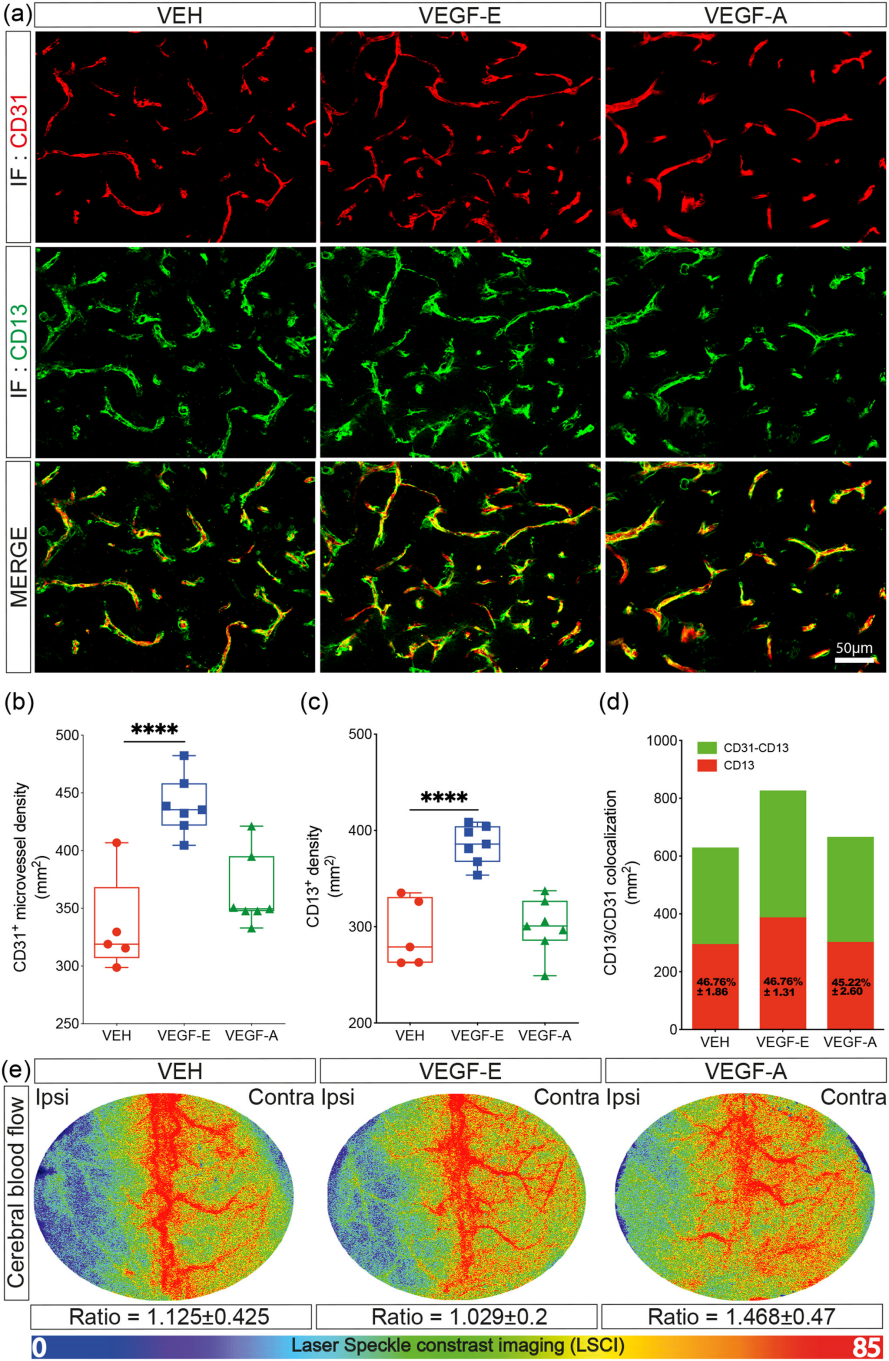
VEGF‐E potently improves vascular density while preserving functionality. (a) Representative fluorescence images of CD31 and CD13 immunolabeling at the injury site in vehicle (VEH)‐, VEGF‐E‐, and VEGF‐A‐treated mice 4 days after stroke. (b) Analysis of the density of CD31^+^ microvessels at the injury site. (c) Analysis of the density of perivascular CD13^+^ cells at the injury site. (d) Analysis of CD31 and CD13 colocalization at the injury site. (e) Representative laser Speckle contrast imaging (LSCI) in VEH‐, VEGF‐E‐, and VEGF‐A‐treated mice at Day 4 after stroke presented as ipsilateral/contralateral ratio of blood flow at Day 4 over 5 min after stroke. Data are boxplot with min/max or stacked histogram (*n* = 5–7 animals/group). ^****^ *p* < 0.0001 compared to control (b, c, one‐way ANOVA). Abbreviations: ANOVA, analysis of variance; IF, immunofluorescence; VEGF, vascular endothelial growth factor; VEH, vehicle.

VEGF‐A‐mediated angiogenesis is associated with an impaired recruitment of pericytes to endothelial cells, which contributes to vascular permeability (Geiseler and Morland 2018; Kim, Cave, and Cho 2021; Zhang et al. 2000). Herein, we aimed to compare the impact of subacute VEGF‐E or VEGF‐A brain delivery via the intranasal route on the structural and functional organization of the vasculature after stroke. This was achieved by immunolabeling CD31 (i.e., mature endothelial cells) and CD13 (i.e., pericytes) at the injury site (Figure 2a). Analysis of CD31 immunolabeling showed that the density of CD31^+^ microvessels at the injury site significantly increased in VEGF‐E‐treated mice in comparison to VEH‐treated mice 4 days after stroke (Figure 2b). Furthermore, VEGF‐E subacute delivery increased the overall density of vascular CD13^+^ cells, which remained unchanged in VEGF‐A‐treated mice (Figure 2c). Notably, VEGF‐E did not compromise the coverage of CD31^+^ microvessels by CD13^+^ cells (Figure 2d), indicating that pericytes are systemically recruited to the newly formed vasculature. Maintaining the interaction of brain endothelial cells with pericytes after ischemic stroke improves brain perfusion (Bernard et al. 2024). Therefore, we investigated next the temporal changes of brain perfusion throughout the subacute phase upon VEGF‐E or VEGF‐A delivery using LSCI. Analysis of the ipsilateral/contralateral ratio of blood flow changes between baseline (prior to treatment) and Day 4 after stroke indicated the brain perfusion in the ipsilateral hemisphere was restored in VEGF‐E‐treated mice (1.029 ± 0.2), outlining improved vascular functions, whereas a cerebral hyperperfusion was reported in VEGF‐A‐treated mice (1.468 ± 0.47), indicative of vascular dysfunction (Figure 2e). These results show that VEGF‐E promotes functional revascularization after stroke associated with preserved coverage with perivascular cells, including pericytes.

### 3.3 VEGF‐E Mediates Stable Neovascularization and Attenuates Neuronal Degeneration

**FIGURE 3:**
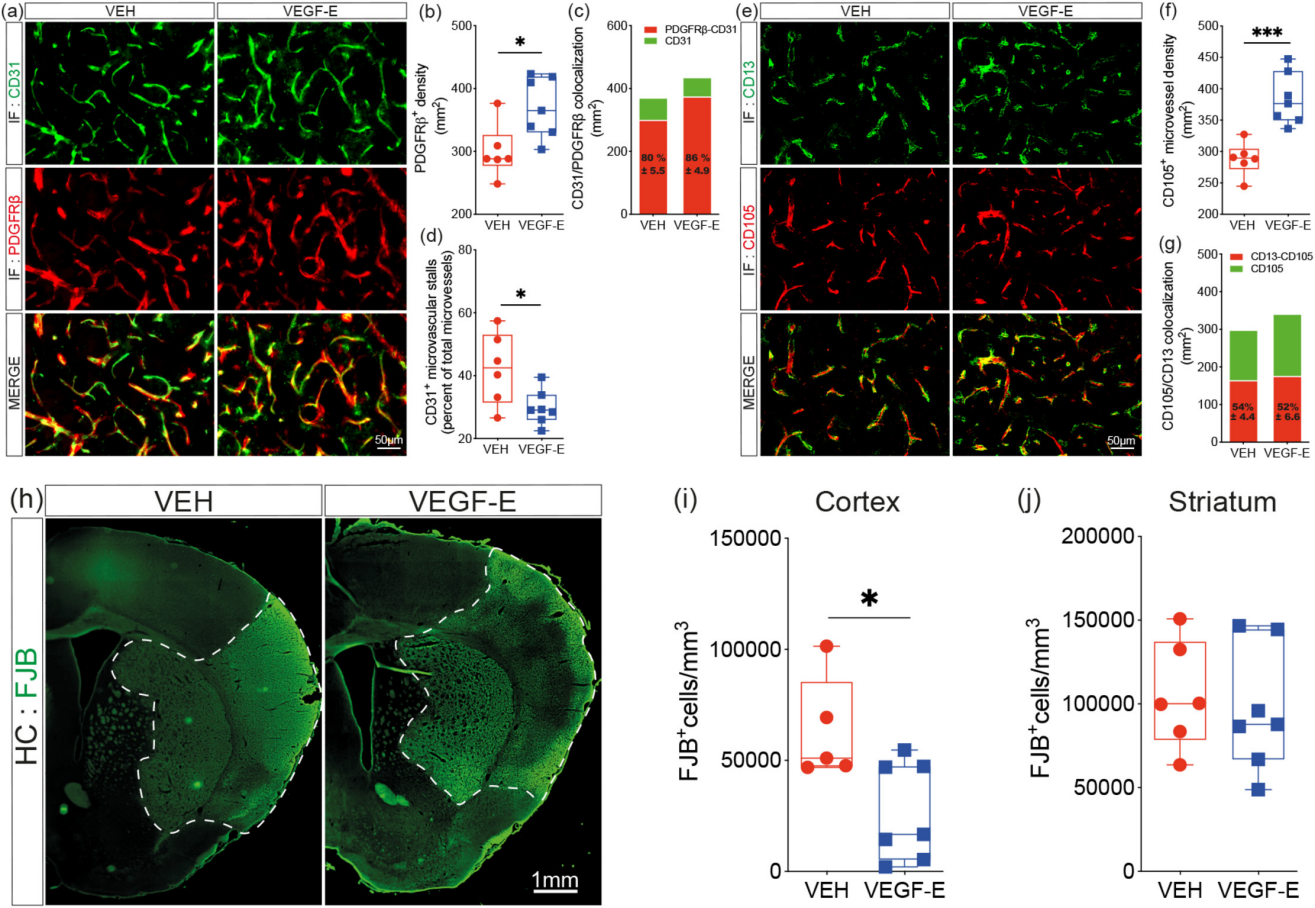
Revascularization is stabilized by VEGF‐E associated with attenuation of neuronal degeneration. (a) Representative fluorescence images of CD31 and PDGFRβ immunolabeling at the injury site in vehicle (VEH)‐ and VEGF‐E‐treated mice 4 days after stroke. (b) Analysis of the density of perivascular PDGFRβ^+^ cells at the injury site 4 days after stroke. (c) Analysis of CD31 and PDGFRβ colocalization at the injury site. (d) Analysis of the number of CD31^+^ microvascular stalls at the injury site. (e) Representative fluorescence images of CD13 and CD105 immunolabeling at the injury site in VEH‐ and VEGF‐E‐treated mice. (f) Analysis of the density of CD105^+^ microvessels at the injury site. (g) Analysis of CD105 and CD13 colocalization at the injury site. (h) Representative fluorescence images of FJB^+^ degenerating neurons in the ipsilateral cortex and ipsilateral striatum. Stereological analysis of the density of FJB^+^ degenerating neurons in the (i) ipsilateral cortex and (j) ipsilateral striatum. Data are boxplot with min/max or stacked histogram (*n* = 6–7 animals/group). *p < 0.05, **p < 0.01, ***p < 0.001 compared to control (b, d, f, i, unpaired two‐tailed *t*‐test). Abbreviations: IF, immunofluorescence; VEGF, vascular endothelial growth factor; VEH, vehicle.

The structural and functional integrity of brain vasculature narrowly depends upon the interaction of brain endothelial cells with perivascular cells (Hermann and ElAli 2012). Our results indicate that VEGF‐E mediates functional revascularization after stroke, more efficiently compared to VEGF‐A. Therefore, we aimed next to further emphasize on exploring the mechanisms underlying VEGF‐E‐mediated effects on vascular structural stabilization and evaluate the consequences on neuronal injury. This was achieved by immunolabeling CD31 (i.e., mature endothelial cells) and PDGFRβ (i.e., all perivascular cells, including pericytes) at the injury site (Figure 3a). VEGF‐E increased the overall density of perivascular PDGFRβ^+^ cells at the injury site 4 days after stroke (Figure 3b) without compromising the coverage of PDGFRβ^+^ cells for CD31^+^ microvessels (Figure 3c). Moreover, VEGF‐E mediated the formation of mature CD31^+^ microvessels associated with reduced number of stalls, indicative of an enhanced vascular patency (Figure 3d). To assess whether the newly formed vessels mediated by VEGF‐E are adequately covered by pericytes, we performed an additional analysis by immunolabeling CD105 (i.e., endoglin) that is expressed in angiogenic endothelial cells (Sier et al. 2021) and CD13 (i.e., pericytes) (Figure 3e). Our analysis indicated that the density of CD105^+^ microvessels upon VEGF‐E delivery increased at the injury site 4 days after stroke (Figure 3f), without compromising the ratio of coverage of CD13^+^ pericytes for CD105^+^ microvessels (Figure 3g). This indicates that the pericytes are actively and rapidly recruited to the nascent angiogenic vasculature to enable stabilization. Finally, we assessed the consequences of VEGF‐E‐mediated functional revascularization on neuronal degeneration using FJB staining (Figure 3h). Our analysis revealed a pronounced reduction of neuronal degeneration in the injured cortex (Figure 3i), a highly vascularized region, but not in the injured striatum (Figure 3j), a less vascularized region, in VEGF‐E‐treated mice at Day 4 after stroke. These results reveal that VEGF‐E promotes stable and functional revascularization, efficiently attenuating neuronal loss during the subacute phase.

### 3.4 VEGF‐E Improves Astrocyte Endfeet Interaction With the Brain Vasculature

**FIGURE 4:**
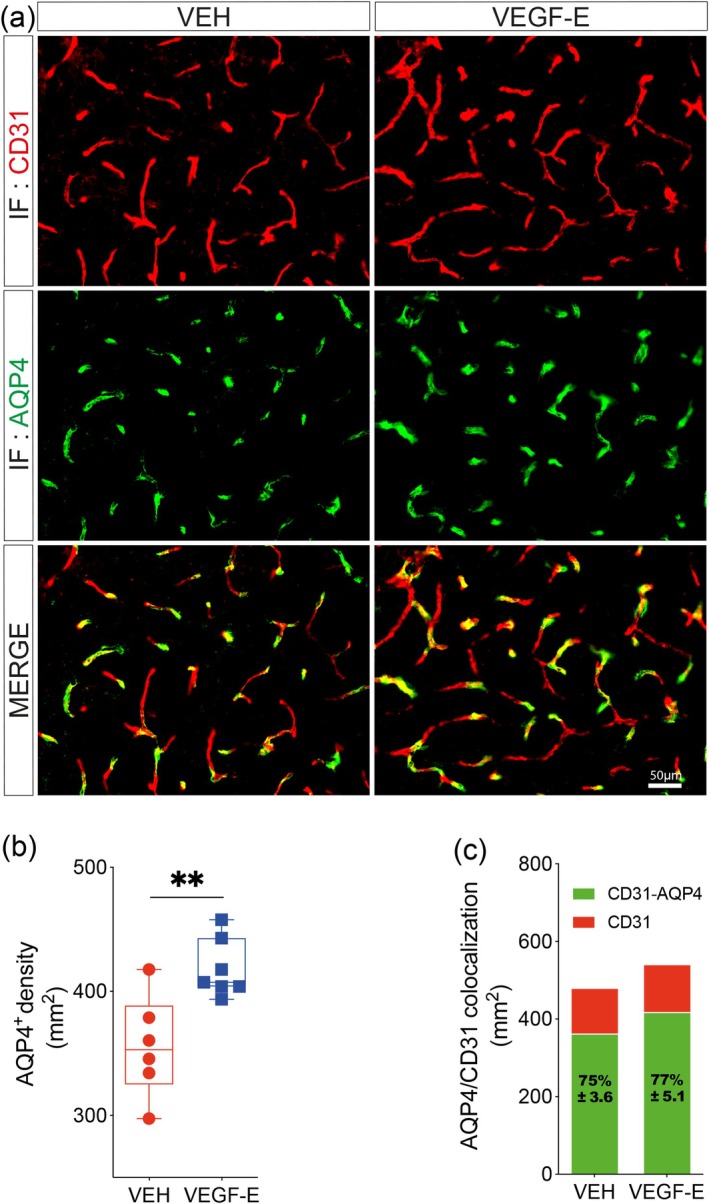
VEGF‐E‐mediated revascularization promotes astrocyte endfeet association. (a) Representative fluorescence images of CD31and AQP4 immunolabeling in the ipsilateral brain in vehicle (VEH)‐ and VEGF‐E‐treated mice 4 days after stroke. (b) Analysis of the density of perivascular AQP4^+^ structures in the ipsilateral hemisphere. (c) Analysis of CD31 and AQP4 colocalization in the ipsilateral hemisphere. Data are boxplot with min/max or stacked histogram (*n* = 6–7 animals/group). **p < 0.01 compared to control (b, unpaired two‐tailed *t*‐test). Abbreviations: IF, immunofluorescence; VEGF, vascular endothelial growth factor; VEH, vehicle.

Endothelial cell interaction with astrocyte endfeet is central to maintain vascular functional properties (Hermann and ElAli 2012). Herein, we analyzed the coverage of the vasculature by astrocyte endfeet by immunolabeling aquaporin 4 (AQP4), and its association with CD31^+^ microvessels at the injury site 4 days after stroke (Figure 4a). Our analysis revealed that the absolute density of AQP4^+^ perivascular structures that wrap brain endothelial cells significantly increased in VEGF‐E‐treated mice compared to VEH‐treated littermates (Figure 4b). Moreover, the density of CD31^+^ microvessels covered by AQP4^+^ perivascular structures was preserved upon subacute VEGF‐E delivery (Figure 4c). These results suggest that the stable revascularization mediated by VEGF‐E improves the association of astrocyte endfeet with brain endothelial cells after ischemic stroke.

### 3.5 Post‐Stroke Astrogliosis Is Attenuated Upon Subacute VEGF‐E Delivery

**FIGURE 5:**
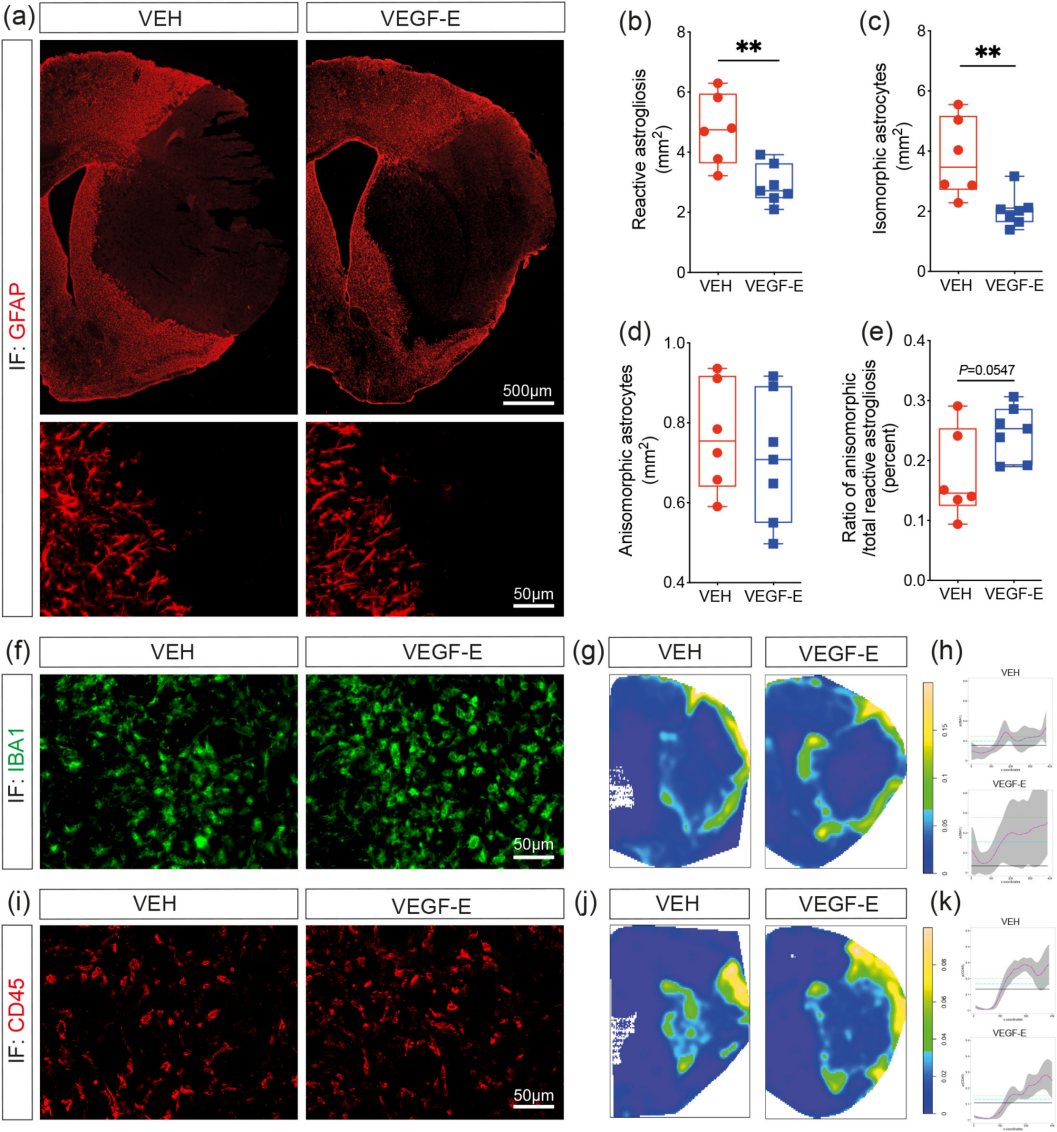
VEGF‐E‐mediated revascularization reduces astrogliosis while slightly increases myeloid cell reactivity. (a) Representative fluorescence images of reactive GFAP^+^ astrocytes (astrogliosis) in the ipsilateral hemisphere and close caption of GFAP immunolabeling in vehicle (VEH)‐ and VEGF‐E‐treated mice 4 days after stroke. (b) Analysis of reactive astrogliosis at the injury site. Analysis of (c) isomorphic, (d) anisomorphic and (e) ratio of anisomorphic astrocytes in total astrogliosis at the GFAP^+^ area in VEH‐ and VEGF‐E treated mice. (f) Representative fluorescence images of IBA1 immunolabeling at the injury site with close caption in VEH‐ and VEGF‐E‐treated mice. (g) PPA of IBA1^+^ cells at the injury site (representing spatial intensity) of VEH‐ and VEGF‐E treated mice. (h) Analysis of the nonparametric estimates of the distribution of IBA1 spatial intensity in respect to hemispheric x‐coordinates in VEH‐ and VEGF‐E treated mice. (i) Representative fluorescence images of CD45 immunolabeling at the injury site with close caption in VEH‐ and VEGF‐E‐treated mice. (j) PPA of CD45^+^ cells at the injury site (representing spatial intensity) of VEH‐ and VEGF‐E‐treated mice. (k) Analysis of the nonparametric estimates of the distribution of CD45 spatial intensity in respect to hemispheric x‐coordinates in VEH‐ and VEGF‐E‐treated mice. Data are boxplot with min/max (*n* = 6–7 animals/group). **p < 0.01 compared to control (b, c, e, unpaired two tailed *t*‐test). Abbreviations: GFAP, glial fibrillary acidic protein; IF, immunofluorescence; PPA, point pattern analysis; VEGF, vascular endothelial growth factor; VEH, vehicle.

Reactive astrocytes play key roles in modulating the immune–vascular interactions after stroke, including immune cell infiltration and glial scar formation (Lawrence et al. 2023; Williamson et al. 2021). Herein, we aimed to investigate the consequences of VEGF‐E‐mediated revascularization on astrocyte reactivity by immunolabeling GFAP (Figure 5a). Our analysis outlined an attenuation of overall astrogliosis translated by a reduced GFAP^+^ reactivity at the injury site in VEGF‐E‐treated mice 4 days after stroke (Figure 5b). Depiction of reactive astrocyte phenotypes indicated that the area occupied by isomorphic GFAP^+^ reactive astrocytes, which are associated with neurodegeneration (Sofroniew 2009), was smaller in VEGF‐E‐treated mice (Figure 5c), while anisomorphic GFAP^+^ reactive astrocytes, which are rather implicated in protective functions (Pekny et al. 2016; Verkhratsky, Rodríguez, and Steardo 2014), remained unchanged (Figure 5d). Notably, VEGF‐E subacute delivery slightly increased the ratio of anisomorphic over isomorphic astrogliosis (Figure 5e). Next, we analyzed the impact of VEGF‐E on microglial cells as well as infiltrating myeloid cells by immunolabeling IBA1 and CD45 4 days after stroke (Figure 5f–k). We calculated a nonparametric estimate of IBA1 spatial intensity (Figure 5f) as a function of x‐coordinates in the ischemic hemisphere (Figure 5g). The logarithmic point process model denoted an increase of 0.23 in spatial intensity in VEGF‐E‐treated mice at random locations of the observed window (Figure 5h). IBA1 is expressed in microglia and infiltrating myeloid cells. Reactive microglia are mainly recruited to the outer side of the injury borderline, whereas infiltrating myeloid cells closely occupy the inner infarct core. Analysis of the spatial intensity of IBA1 outlined a preferential recruitment of reactive microglia as well as infiltrated myeloid cells to the peri‐infarct region that is undergoing angiogenesis. Analysis of the spatial intensity of CD45^+^ cells using the logarithmic point process model (Figure 5i, j) indicated that at a given location, VEGF‐E‐treated mice displayed a slightly higher CD45 spatial intensity (0.29) compared to VEH‐treated littermates (Figure 5k). These results indicate that VEGF‐E‐mediated revascularization is associated with a reduction in detrimental astrogliosis and localization of reactive myeloid cells to the peri‐infarct.

### 3.6 VEGF‐E Mediates the Endothelial Release of Factors Promoting Perivascular Cell Functions

**FIGURE 6:**
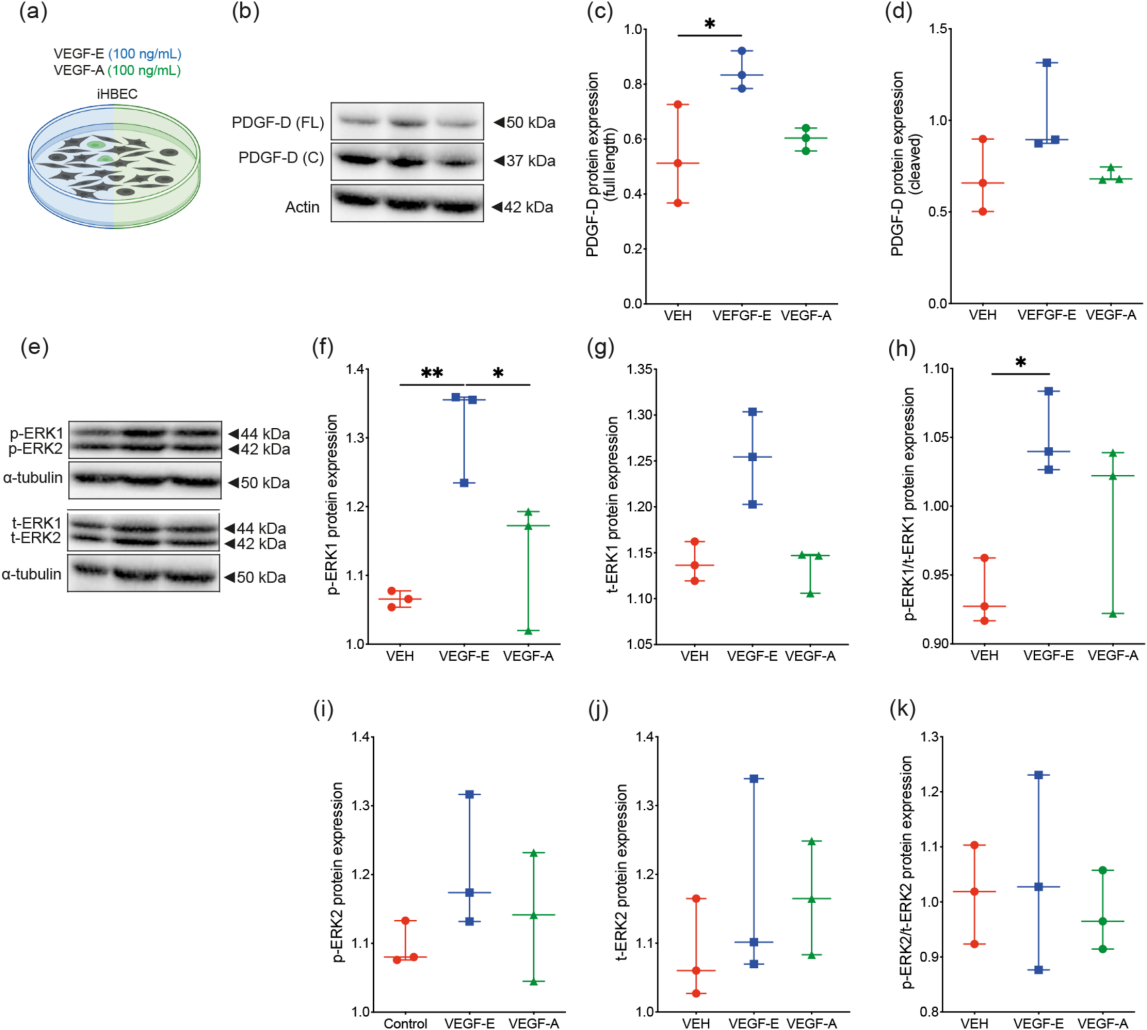
VEGF‐E increases basal PDGF‐D expression in brain endothelial cells by activating ERK1/2 pathway. (a) Schematic illustration of the experimental design of iHBEC stimulated with vehicle (VEH), VEGF‐E (100 ng/mL), or VEGF‐A (100 ng/mL) for 24 h, repeated for another 24 h under normoxic condition. (b) Representative Western blot images of PDGF‐D expression in iHBEC upon stimulation with VEH, VEGF‐E, or VEGF‐A. Analysis of the expression of (c) the full length (FL) and (d) the cleaved form of PDGF‐D. (e) Representative Western blot images of phosphorylated (p) and total (t) ERK1/2 expression in iHBEC upon stimulation with VEH, VEGF‐E, or VEGF‐A. Analysis of (f) p‐ERK1 and (g) t‐ERK1 expression as well as (h) p‐ERK1/t‐ERK1 ratio (i.e., pathway activation). Analysis of (i) p‐ERK2 and (j) t‐ERK2 expression as well as (k) p‐ERK2/t‐ERK2 ratio (i.e., pathway activation) in iHBEC upon stimulation with VEH, VEGF‐E, or VEGF‐A. Data are boxplot with min/max (*n* = 3 independent experiments/condition). *p < 0.05/**p < 0.01 compared to control (c, f, h, one‐way ANOVA). Abbreviations: ANOVA, analysis of variance; iHBEC, immortalized human brain endothelial cells; PDGF, platelet‐derived growth factor; VEGF, vascular endothelial growth factor; VEH, vehicle.

Endothelial cells secrete PDGF ligands that bind and activate PDGFRβ to promote the recruitment of perivascular cells, mainly pericytes, to nascent endothelial sprouts, a crucial process for vascular stabilization (Arimura et al. 2012). Our group has recently demonstrated that PDGF‐D, which specifically binds to PDGFRβ, plays a major role in stabilizing the angiogenic vasculature after stroke by rescuing the functions of pericytes and crosstalk with brain endothelial cells. Our findings indicated that VEGF‐E improved the interaction of brain endothelial cells with perivascular cells, which was less efficient with VEGF‐A. Therefore, we assessed next the expression of PDGF‐D in brain endothelial cells (i.e., iHBEC) exposed to an angiogenic context via stimulation with VEGF‐E (100 ng/mL) or VEGF‐A (100 ng/mL) using cell‐based assays (Figure 6a). Western blot analysis (Figure 6b) revealed that expression of the full‐length form of PDGF‐D, which represents the intracellular form prior to extracellular release, was potently increased upon VEGF‐E stimulation, but not VEGF‐A (Figure 6c). Likewise, expression of the cleaved form of PDGF‐D, which represents the released form, was increased upon VEGF‐E stimulation (Figure 6d), but to a lesser extent compared to the full length form, probably due to its increased release into the extracellular space upon VEGF‐E stimulation. Notably, previous reports have demonstrated that ERK1/2 pathway plays a central role in regulating the expression of PDGF‐D, which contributes to the differentiation of endothelial cells (Lu et al. 2022). Western blot analysis (Figure 6e) showed that VEGF‐E, and not VEGF‐A, increased ERK1 phosphorylation (p‐ERK1) (Figure 6f) and total expression (t‐ERK1) (Figure 6g). Notably, VEGF‐E stimulation enhanced the ratio of phosphorylated/total ERK1 (Figure 6h), highlighting ERK1 pathway activation. The effects of VEGF‐E on ERK2 pathway were less pronounced (Figure 6i–k). These results show that VEGF‐E induces the expression of PDGF‐D in brain endothelial cells via ERK1 pathway activation to promote perivascular cell recruitment.

### 3.7 VEGF‐E‐Mediated Activation of Brain Endothelial Cells Stimulates Perivascular Cell Mobility

**FIGURE 7:**
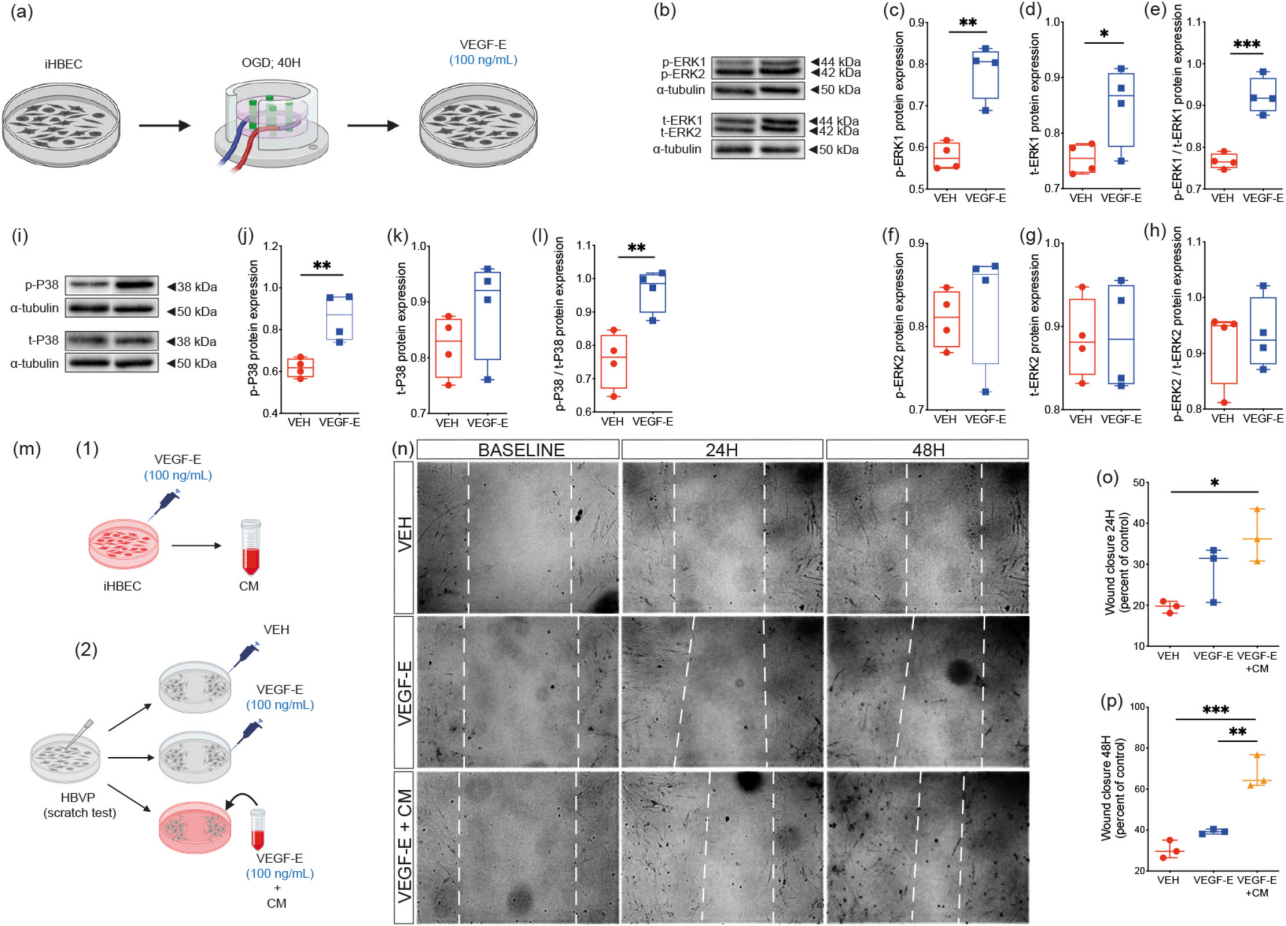
VEGF‐E‐stimulated brain endothelial cells promote perivascular cell mobility under ischemia and reperfusion‐like condition. (a) Schematic illustration of the experimental design of iHBEC exposed to OGD and reperfusion followed by stimulation with vehicle (VEH) or VEGF‐E (100 ng/mL) for 24 h. (b) Representative Western blot images of phosphorylated (p) and total (t) ERK1/2 expression in iHBEC stimulated with vehicle (VEH) or VEGF‐E. Analysis of (c) p‐ERK1 and (d) t‐ERK1 expression as well as (e) p‐ERK1/t‐ERK1 ratio (i.e., pathway activation). Analysis of (f) p‐ERK2 and (g) t‐ERK2 expression as well as (h) p‐ERK2/t‐ERK2 ratio (i.e., pathway activation) in iHBEC stimulated with VEH and VEGF‐E after OGD. (i) Representative Western blot images of phosphorylated (p) and total (t) P38 expression. Analysis of (j) p‐P38 and (k) t‐P38 expression as well as (l) p‐P38/t‐P38 ratio (i.e., pathway activation) in iHBEC stimulated with VEH and VEGF‐E after OGD. (m) Schematic illustration of the experimental design of wound healing assay performed on HBVP after stimulation with VEH, VEGF‐E, or the conditioned medium (CM) of VEGF‐E‐treated iHBEC. (n) Representative brightfield images of the wound in VEH, VEGF‐E, or CM‐stimulated HBVP at baseline, 24 h, or 48 h. Analysis of wound closure of VEH, VEGF‐E, or CM‐treated HBVP at (o) 24 h and (p) 48 h, represented as percent of control (baseline, 0 h). Data are boxplot with min/max (n = 3–4 independent experiments/condition). *p < 0.05/**p < 0.01/***p < 0.001 compared to control (c‐e, j, l, unpaired two‐tailed *t*‐test; o, p, one‐way ANOVA). Abbreviations: H, hours; iHBEC, immortalized human brain endothelial cells; OGD, oxygen and glucose‐deprived; VEGF, vascular endothelial growth factor; VEH, vehicle.

Our hereabove findings suggest that unlike VEGF‐A, VEGF‐E mediates an angiogenic response in brain endothelial cells while promoting vascular stabilization via the release of factors that improve the recruitment of perivascular cells. Therefore, we aimed next to further explore the role of VEGF‐E in regulating brain endothelial cell capacity to mobilize perivascular cells under ischemia and reperfusion‐like conditions. First, brain endothelial cells (i.e., iHBEC) were exposed to OGD followed by reperfusion and subsequently stimulated with VEGF‐E (100 ng/mL) (Figure 7a). We first assessed the activation of ERK1/2 and P38 MAPK pathway, which play an important role in regulating the angiogenic response in brain endothelial cells (Gee, Milkiewicz, and Haas 2010). Western blot analysis (Figure 7b) indicated that VEGF‐E potently increased ERK1 phosphorylation (p‐ERK1) (Figure 7c) and total expression (t‐ERK1) (Figure 7d), as well as enhanced the ratio of phosphorylated/total ERK1 (Figure 7e) in iHBEC exposed to OGD, translating an activation of ERK1 pathway. As shown above, VEGF‐E effects on ERK2 pathway were less potent, translated by an unchanged phosphorylation (p‐ERK2) (Figure 7f), total expression (t‐ERK2) (Figure 7g), and ratio of phosphorylated/total ERK2 (Figure 7h). Furthermore, Western blot analysis (Figure 7i) showed that VEGF‐E stimulation increased P38 phosphorylation (p‐P38) (Figure 7j), without affecting its total expression (Figure 7k), while increasing the ratio of phosphorylated/total P38 (Figure (7l)), outlining an enhanced activation of P38 MAPK pathway. The increased expression and release of PDGF‐D in VEGF‐E‐stimulated iHBEC could potentially underlie the enhanced association of perivascular cells with brain endothelial cells after stroke. Therefore, we aimed next to further explore whether the secretome of VEGF‐E‐stimulated iHBEC could affect the dynamics of perivascular cells. For this purpose, we used the wound healing paradigm to evaluate the impact of iHBEC stimulation with VEGF‐E on the migratory capacity of brain perivascular cells (i.e., HBVP), which is essential for the recruitment to brain endothelial cells (Figure 7m). HBVP were either directly stimulated with VEGF‐E or with the conditioned medium (CM) of iHBEC previously stimulated with VEGF‐E (Figure 7n). Our results showed that wound closure was significantly improved upon exposure of HBVP for 24 h with CM (Figure 7o), an effect that was further accelerated upon prolonged exposure for 48 h (Figure 7p), outlining an enhanced migratory capacity. Direct stimulation of HBVP to VEGF‐E alone did not affect wound closure, indicating that VEGF‐E‐mediated effects on HBVP migration were driven by factors released by brain endothelial cells, including PDGF‐D. These results show that VEGF‐E promotes the angiogenic response in brain endothelial cells, while preserving stability meditated by released mediators that stimulate the functions of perivascular cells.

## 4 Discussion

Upon ischemic stroke, a potent VEGF‐A mediated angiogenic response is triggered at the injury site to increase vascular density as an attempt to restore brain perfusion and eliminate cell debris by immune cells (Jean LeBlanc, Guruswamy, and ElAli 2018; Hermann and Zechariah 2009). Therefore, it was proposed that exogenous promotion of revascularization represents a promising therapeutic avenue for ischemic stroke (Navaratna et al. 2009). However, most of the investigations that have assessed the therapeutic potential of VEGF‐A led to mitigated results (Hermann and Zechariah 2009; Hu et al. 2022; Lange et al. 2016). Indeed, exogenous administration VEGF‐A constitutes a double‐edged sword strategy, as it significantly increases the risk of vascular destabilization and hemorrhagic transformation (Hermann and Zechariah 2009; Hu et al. 2022; Lange et al. 2016). These detrimental effects have dampened the translational potential of VEGF‐A‐mediated revascularization in ischemic stroke therapy. VEGF‐E, a non‐mammalian VEGF‐A encoded by the parapox Orf virus, has been reported to stimulate the healing of cutaneous lesions by promoting potent and stable vascularization while attenuating inflammation, without compromising permeability (Wise et al. 2003, 2020). The therapeutic promises of VEGF‐E in ischemic stroke remain completely unexplored. Herein, using an experimental ischemic stroke model combined with cell‐based assays, our investigations shed the light onto the role of VEGF‐E in mediating reparative revascularization after ischemic stroke. Our findings indicate that VEGF‐E non‐invasive subacute brain delivery improves neurological recovery and attenuates injury progression by promoting the formation of stable and functional vasculature after ischemic stroke. Notably, VEGF‐E increases mature microvessel density at the injury site, while promoting stability via preservation of brain endothelial cell interaction with pericytes, which is compromised with VEGF‐A. Moreover, we reveal that VEGF‐E‐mediated revascularization is associated with a normal restoration of blood flow in the ipsilateral hemisphere, whereas VEGF‐A‐mediated revascularization corroborates with an abnormal increase of blood flow, outlining vascular dysfunction. Furthermore, VEGF‐E‐mediated effects are associated with a reduced astrogliosis. Our cell‐based assays demonstrate that VEGF‐E triggers the activation of ERK‐1/2 pathway more potently compared to VEGF‐A, as well as P38 pathway, which are both key components of VEGFR‐2 signaling. Interestingly, we reveal that VEGF‐E increases the expression and release of PDGF‐D that rescues the survival and functions of PDGFRβ^+^ perivascular cells, including pericytes. Indeed, we report that the secretome of VEGF‐E‐stimulated brain endothelial cells enhances pericyte mobility that is required for recruitment to the vasculature. Our study outlines the potential of VEGF‐E in improving subacute recovery after stroke by promoting stable and functional revascularization.

Deregulation of the vascular structure and functions contributes to secondary injury in the subacute phase, worsening stroke outcomes (Duan et al. 2024; Gao et al. 2023; Goodman et al. 2023; X. Hu et al. 2017). Herein, we show that unlike VEGF‐A, VEGF‐E increases vascular density without comprising permeability, translated by a reduced extravasation of IgG. VEGF‐E improves the recovery of motor functions more efficiently compared to VEGF‐A. These observations suggest that VEGF‐E‐mediated revascularization initiated as early as 24 h after stroke achieves adequate structural and functional protection, whereas, in line with previous reports, administration of VEGF‐A within 24 h after ischemic stroke compromises vascular stability (Hu et al. 2022; Zhang et al. 2000). Angiogenesis is a narrowly controlled process that comprises intimate crosstalk between brain endothelial cells and perivascular cells (Fang, Wang, and Miao 2023; Winkler, Bell, and Zlokovic 2011). Upon ischemic stroke, the newly formed microvessels are often unstable due to inadequate recruitment of perivascular cells, mainly pericytes, as well as astrocyte endfeet (Fang, Wang, and Miao 2023; Winkler, Bell, and Zlokovic 2011). VEGF‐A mediates its angiogenic action by acting on VEGFR‐2, which is abundantly expressed in endothelial cells (Lange et al. 2016). VEGF‐E specifically binds and activates VEGFR‐2 with the same affinity as VEGF‐A (Ogawa et al. 1998; Pérez‐Gutiérrez and Ferrara 2023). Evidence is indicating that VEGF‐E and VEGF‐A activate overlapping as well as distinct downstream signaling pathways in brain endothelial cells, probably due to the fact that VEGF‐A binding to VEGFR‐2 involves the co‐receptor neuropilin‐1, but not VEGF‐E (Hudson et al. 2014; Ogawa et al. 1998; Pérez‐Gutiérrez and Ferrara 2023). In contrast to VEGF‐A, VEGF‐E does not bind to VEGFR‐1, which acts as a decoy receptor in endothelial cells to fine‐tune VEGFR‐2 signaling via modulation of VEGF‐A bioavailability, but as a signaling receptor in perivascular cells and immune cells (Ogawa et al. 1998; Pérez‐Gutiérrez and Ferrara 2023). Notably, VEGF‐A binds VEGFR‐1 with higher affinity compared to VEGFR‐2, indicating that VEGF‐E bioavailability to bind VEGFR‐2 is more important (Hudson et al. 2014; Ogawa et al. 1998; Pérez‐Gutiérrez and Ferrara 2023). Herein, we show that VEGF‐E potently increases the density of mature CD31^+^ microvessels while preserving the recruitment of perivascular PDGFRβ^+^ cells, including CD13^+^ pericytes, to brain endothelial cells, which may account for the improved vascular stability. Furthermore, we show that the increased vascular density mediated by VEGF‐A, and not by VEGF‐E, is accompanied by a cerebral hyperperfusion in the ipsilateral hemisphere. Deregulation of neurovascular coupling associated with alteration of blood flow autoregulation upon brain injuries has been shown to lead to cerebral hyperperfusion, which increases intracranial pressure and subsequently worsening outcomes (Toth et al. 2016). This further confirms that unlike VEGF‐A, VEGF‐E mediates the formation of functional mature vasculature after ischemic stroke, potentially by rescuing the functions of pericytes. Notably, the increased vascular density upon VEGF‐E subacute delivery exhibits reduced number of stalls, which are caused by impaired vascular functions due to pathological contraction of pericytes (Shrouder et al. 2024). This outlines an improved patency of the vasculature, which is corroborated by restoration of blood flow. Interestingly, rescuing the survival and functions of perivascular cells, mainly pericytes, significantly improves vascular potency and brain perfusion after stroke (Bernard et al. 2024). Notably, VEGF‐E increases the density of CD105^+^ angiogenic active microvessels at the injury site, without compromising the coverage with CD13^+^ pericytes. CD105 is a transmembrane glycoprotein that binds transforming growth factor‐β1/3 (TGF‐β1/3) (Warrington et al. 2005; Zhu et al. 2017) and is abundantly expressed in proliferating endothelial cells during angiogenesis (Rossi, Bernabeu, and Smadja 2019; Zhu et al. 2017). CD105 plays an important role in regulating endothelial–perivascular cell interaction, which is required to maintain vascular integrity (Sweeney and Foldes 2018). This suggests that VEGF‐E stimulates a rapid recruitment of pericytes to the newly formed vasculature, potentially accounting for the improved stability. The coverage of microvessels by astrocyte endfeet expressing AQP4 is essential for vascular stability and BBB functions (Mathiisen et al. 2010; Williamson et al. 2021; Zhou et al. 2008). AQP4 perivascular expression is reduced at the injury site after ischemic stroke (Friedman et al. 2009; Gu et al. 2022). Herein, we report that the density of CD31^+^ mature microvessels covered by AQP4^+^ astrocyte‐endfeet remains elevated upon VEGF‐E delivery. These observations suggest that VEGF‐E increases vascular density after stroke without comprising structural and functional integrity via adequate association with perivascular cells and astrocyte endfeet. These observations are in line with previous reports showing that VEGF‐E triggers structurally stable cutaneous vascularization (Kiba et al. 2003; Wise et al. 2018). Furthermore, VEGF‐E‐mediated revascularization is associated with a reduced density of degenerating neurons, outlining an attenuation of secondary injury progression after stroke.

Inflammation plays a key role in the pathobiology of ischemic stroke (Anrather and Iadecola 2016; Jayaraj et al. 2019; Jiang et al. 2021). Brain injury induces the activation of astrocytes and microglial cells, which contribute to glial scar formation that aims to isolate the injury site and partake in tissue repair (ElAli and Jean LeBlanc 2016; He, Yang, and Zhang 2022; Lampron, ElAli, and Rivest 2013; Manrique‐Castano and ElAli 2021; Shen et al. 2021). As mentioned, VEGF‐A binds VEGFR‐1 that acts as a signaling receptor in various cells, including glial cells and immune cells (Tchaikovski, Fellbrich, and Waltenberger 2008; Uemura et al. 2021). Indeed, VEGFR‐1 is abundantly expressed in monocytes that infiltrate the injury site after ischemic stroke to differentiate into microglial‐like cells (ElAli and Jean LeBlanc 2016; Lampron, ElAli, and Rivest 2013), regulating VEGF‐A‐mediated chemotactic responses (Tchaikovski, Fellbrich, and Waltenberger 2008). Deregulation of astroglial cell reactivity and excessive microglial cell activation exacerbate injury severity after ischemic stroke (Liang et al. 2023). Previous reports have indicated that during central nervous system (CNS) angiogenesis, activation of VEGFR‐1 increases vascular permeability and inflammation by promoting a pro‐inflammatory phenotype in microglial cells (Uemura et al. 2021). Our findings show that VEGF‐E administration reduces the isomorphic reactivity of GFAP^+^ astrocytes, which are associated with neurodegeneration, while maintaining the anisomorphic reactivity of GFAP^+^ astrocytes, which are implicated in segregating the injured tissue to protect the intact region (Muñoz‐Ballester and Robel 2023; Pekny et al. 2016). Infiltrating monocyte‐derived microglial cells contribute to neurovascular repair after ischemic stroke via various processes, including phagocytosis of necrotic and apoptotic cells (ElAli and Jean LeBlanc 2016; Gliem et al. 2012). The density of CD45^+^ cells as well as IBA1^+^ cells, which comprise microglia and infiltrating myeloid cells, slightly increases upon VEGF‐E delivery. Alteration of monocyte recruitment to the injured brain after stroke impairs angiogenesis and functional recovery (Pedragosa et al. 2020). As VEGF‐E does not act on VEGFR‐1, it is presumable that the enhanced activation of microglial cells is involved in protective functions, including the removal of degenerative neurons and fine‐tuning the angiogenic response (Pedragosa et al. 2020). Additional research is required to fully elucidate the contribution of microglial cells to the VEGF‐E‐mediated revascularization after stroke, especially that it does implicate VEGFR‐1. This indicates that VEGF‐E mediates an efficient revascularization after ischemic stroke and attenuates glial cell reactivity without accentuating detrimental neuroinflammation.

VEGFR‐2 mediates its angiogenic effects by activating various downstream intracellular signals, including ERK1/2 and P38 pathways, to regulate key processes in endothelial cells, such as migration, survival, and proliferation (Lange et al. 2016; Pérez‐Gutiérrez and Ferrara 2023). Notably, VEGF‐E induces angiogenesis faster than VEGF‐A by stimulating endothelial cells without involving neuropilin‐1, a co‐receptor that promotes VEGF‐A binding to VEGFR‐2 (Kiba et al. 2003; Zheng et al. 2007). This could potentially account for the different downstream signals that could operate in response to VEGF‐E. Notably, we show that VEGF‐E stimulation potently stimulates the activation of ERK1/2 and P38 pathways in human brain endothelial cells. Our results are in line with recent reports showing that VEGF‐E alleviates brain endothelial cell apoptosis upon hypoxia via regulation of ERK1/2 pathway, subsequently preserving barrier properties (J. Wang et al. 2024). These observations underlie VEGF‐E‐mediated potent angiogenesis without compromising the barrier functions of brain endothelial cells, which could, at least partially, explain the reduced extravasation of IgG. Our observations are in line as well with previous findings demonstrating that VEGF‐E induces a stable and non‐edematous vascularization in cutaneous lesions (Kiba et al. 2003). VEGF‐E does not bind to PDGFRs, including PDGFRα expressed essentially in fibroblasts as well as glial cells, and PDGFRβ expressed mostly in perivascular cells (Shibuya 2011). This suggests that the improved vascular coverage of perivascular cells upon VEGF‐E stimulation is mediated by brain endothelial cell‐dependent mechanisms. Indeed, we show that unlike VEGF‐A, VEGF‐E increases the expression and release of PDGF‐D by brain endothelial cells. PDGF‐D has been recently identified as a specific ligand of PDGFRβ (Reigstad, Varhaug, and Lillehaug 2005). In perivascular cells, PDGFRβ plays critical roles in regulating survival, migration, and recruitment to endothelial cells (Winkler, Bell, and Zlokovic 2010). Our group has recently demonstrated that PDGF‐D is induced in angiogenic brain endothelial cells and contributes to the preservation of neurovascular functions mainly by rescuing the survival and functions of perivascular cells and association with the vasculature (Bernard et al. 2024). Moreover, previous reports have shown that ERK1/2 pathway drives PDGF‐D expression to promote the differentiation of brain endothelial cells (Lu et al. 2022). Herein, we report that VEGF‐E potently activates ERK1/2 pathway in brain endothelial cells and induces the expression and release of PDGF‐D that acts on neighboring perivascular cells. Indeed, we show that the secretome of human brain endothelial cells stimulated with VEGF‐E, and not VEGF‐E itself, potently promotes the migratory capacity of human brain pericytes exposed to ischemia and reperfusion‐like condition. This potentially accounts for the enhanced vascular recruitment of PDGFRβ^+^ perivascular cells. These observations are in line with previous reports demonstrating that in cutaneous lesions, VEGF‐E induces a well‐organized vascular network associated with an enhanced recruitment of pericytes (Kiba et al. 2003). These results demonstrate that VEGF‐E promotes brain endothelial cell angiogenic response while maintaining structural and functional barrier functions and stimulating the recruitment of perivascular cells.

Herein, we provide evidence that unlike VEGF‐A, VEGF‐E enables reparative revascularization of the injured tissue after ischemic stroke. Our study demonstrates that VEGF‐E‐mediated functional revascularization represents a novel therapeutic approach to efficiently attenuate secondary injury progression and to subsequently ameliorate subacute neurological recovery after ischemic stroke.

## Author Contributions

**Romain Menet:** data curation, formal analysis, methodology, validation, visualization, writing – original draft, writing – review and editing. **Leila Nasrallah:** data curation, formal analysis, methodology, validation, visualization, writing – original draft, writing – review and editing. **Maxime Bernard:** data curation, formal analysis, methodology, validation, visualization, writing – original draft, writing – review and editing. **Anne‐Sophie Allain:** formal analysis, methodology. **Ayman ElAli:** conceptualization, data curation, formal analysis, funding acquisition, project administration, resources, supervision, writing – original draft, writing – review and editing.

## Conflicts of Interest

The authors declare no conflicts of interest.

### Peer Review

The peer review history for this article is available at https://www.webofscience.com/api/gateway/wos/peer‐review/10.1111/ejn.70114.

## Data Availability

All other data are available from the corresponding author upon reasonable request.
